# SynRXN: An Open Benchmark and Curated Dataset for Computational Reaction Modeling

**DOI:** 10.1038/s41597-026-07260-w

**Published:** 2026-04-20

**Authors:** Tieu-Long Phan, Nhu-Ngoc Nguyen Song, Peter F. Stadler

**Affiliations:** 1 Bioinformatics Group, Department of Computer Science & Interdisciplinary Center for Bioinformatics & School for Embedded and Composite Artificial Intelligence (SECAI), Leipzig University, Härtelstraße 16-18, D-04107 Leipzig, Germany; 2 Department of Mathematics and Computer Science, University of Southern Denmark, DK-5230 Odense M, Denmark; 3 School of Pharmacy, University of Medicine and Pharmacy at Ho Chi Minh City, Dinh Tien Hoang, Ho Chi Minh City, Vietnam; 4 Max Planck Institute for Mathematics in the Sciences, Inselstraße 22, D-04103 Leipzig, Germany; 5 Department of Theoretical Chemistry, University of Vienna, Währingerstraße 17, A-1090 Vienna, Austria; 6 Facultad de Ciencias, Universidad National de Colombia, Bogotá, Colombia; 7 Center for non-coding RNA in Technology and Health, University of Copenhagen, Ridebanevej 9, DK-1870 Frederiksberg, Denmark; 8 Santa Fe Institute, 1399 Hyde Park Rd., Santa Fe, NM 87501 USA

## Abstract

We present SynRXN, a unified benchmark dataset resource for computer-aided synthesis planning (CASP). SynRXN decomposes end-to-end synthesis planning into five task families, covering reaction rebalancing, atom-to-atom mapping, reaction classification, reaction property prediction, and synthesis prediction. Curated, provenance-tracked reaction corpora are assembled from heterogeneous public sources into a harmonized representation and packaged as versioned datasets for each task family, with explicit source metadata, licence tags, and machine-readable manifests that record checksums and row counts. For every task, SynRXN provides predefined, leakage-aware partitions, and standardized evaluation metrics tailored to classification, regression, and structured prediction settings. For sensitive benchmarking, we combine public training and validation data with held-out gold-standard test sets, and contamination-prone tasks such as *reaction rebalancing* and *atom-to-atom mapping* are distributed only as evaluation sets and are explicitly not intended for model training. Scripted build recipes enable bitwise-reproducible regeneration of all corpora across machines and over time, and the entire resource is released under permissive open licences to support reuse and extension. By removing dataset heterogeneity and packaging transparent, reusable benchmark specifications, SynRXN enables fair longitudinal comparison of CASP methods, supports rigorous ablations and stress tests along the full reaction-informatics pipeline, and lowers the barrier for practitioners who seek robust and comparable performance estimates for real-world synthesis planning workloads.

## Background & Summary

Computer-aided synthesis planning (CASP) assists chemists in designing feasible synthetic routes by combining mechanistic insight, experimental data, and algorithmic search. In parallel with model innovations for reaction prediction and retrosynthesis^1–4^, the field has been accelerated by the emergence of standardized open repositories and newly curated reaction databases^5^ that enable large-scale, higher-quality training and evaluation. These resources have supported improved reaction prediction and retrosynthesis as well as more effective multi-step route design^6–9^. We decouple CASP into three branches according to the *chemical object being modeled* and the corresponding input to output transformation, namely *reaction curation*^10^, *reaction characterization*^11^, and *synthesis prediction*^1^. In *reaction curation*, heterogeneous reaction extractions are converted into chemically executable and template ready records by repairing incomplete equations such as missing counter ions, reagents, or coproducts, restoring mass balance, and producing atom-to-atom maps (AAM) that localize bond changes for reaction center identification and template extraction. Given curated records, *reaction characterization* assigns chemically meaningful identities and attributes to transformations such as reaction class or role labels and physicochemical properties. Finally, *synthesis prediction* models single-step chemistry via forward reaction prediction and single-step retrosynthesis. We omit multi-step route planning because route level outcomes depend strongly on stock definitions and search heuristics instead of isolated model performance^12,13^.

Anchoring the reaction curation phase, the pipeline begins with raw reaction data, which are often noisy or incomplete. Extractions from patents, electronic lab notebooks (ELNs), and the literature can omit solvents, counterions, stoichiometric reagents, or byproducts and can contain inconsistent stoichiometry or charge assignments^10^. Such errors corrupt model inputs and bias learned representations^14^. Automated *reaction rebalancing* methods^15^ restore elemental and charge balance using rule-based and graph-based corrections, thereby producing cleaner inputs for subsequent stages. Because rebalancing is a corrective preprocessing step, standardized rebalancing test sets and diagnostics are necessary to quantify correction accuracy and to assess how residual inconsistencies may influence downstream tasks.

To complete the reaction curation process once stoichiometry is addressed, AAM establishes the structural lineage that reveals the microscopic changes defining each transformation. Accurate AAM is essential for identifying reaction centers, extracting mechanistic templates, and supervising models that reason about bond changes. Foundational studies have established robust automated mapping methods for complex reactions^16^, and contemporary toolchains include transformer-based mappers such as RXNMapper^17^, graph-based mappers such as GraphormerMapper^18^ and LocalMapper^19^, and heuristic mappers such as Indigo^20^ and RDTool^21^. Ensemble strategies that arbitrate among multiple mappers^22,23^ increase coverage and flag low-confidence correspondences. Because mapping errors propagate into template extraction and mechanistic features, AAM benchmarking should ideally rely on curated, held-out gold standards and report exact match accuracy. Where such standards are unavailable, consensus among multiple mapping tools can serve as a proxy for identifying high-confidence subsets^23^. It should be kept in mind, however, that this approach does not replace the need for human-verified validation.

For reaction characterization, models assign taxonomic identity to these standardized records resulting from structural curation and template extraction. When accurate atom-to-atom mappings are available, template extraction and mechanistic clustering are straightforward. Because AAM are not universally available or reliable^16^ for many corpora, reaction classification performed without atom mapping is still of practical importance and widely used. Benchmarks, therefore, should evaluate both regimes and their sensitivity to mapping quality. *Reaction classification* groups transformations by mechanism, functional-group change, or *named-reaction* taxonomy, and supports search, curation, and downstream prediction. Methods range from engineered fingerprints and compact differential descriptors to learned embeddings. Engineered fingerprints enabled the first large-scale categorization efforts^24^. The compact, alignment-free descriptor DRFP encodes bond-change information via hashed circular substructure differences and remains competitive in small-data, interpretable settings^11^. The learned representation RXNFP derives attention-based reaction embeddings from Molecular Transformer sequence models^25^. Molecule-level cross-attention GNNs such as SynCat explicitly model intermolecular context and reagent roles^26^. Consequently, classification benchmarks require representative label taxonomies and transparent splitting procedures, together with repeated resampling to reveal robustness, calibration, and per-class behavior across method families.

Beyond categorical labels, *reaction property prediction* targets continuous and probabilistic quantities that guide experimental decisions. These include yields and physicochemical properties such as activation barriers and transition-state features. Graph- and sequence-based deep models have shown promise for yield and condition recommendations^27,28^, and representation-learning approaches (e.g., *Condensed Graph of Reactions*^29^ or *Imaginary Transition State*^30^ embeddings) extend to thermochemical and kinetic targets^31^. Public barrier corpora such as QMrxn/QMrxn20^32^ and curated SN2/E2 collections, as well as community benchmarking suites (e.g., Chemprop datasets^33^) provide training and test splits for systematic barrier modeling and evaluation^34^. These studies show that machine learning predictors can approximate higher level quantum calculations when trained on high quality labels. However, they also reveal limited out-of-distribution transferability for transition-state properties, which motivates the use of QM-augmented descriptors and transition-state-based architectures^31,34^.

The final stage is *synthesis prediction*, which composes single-step predictions into multi-step routes under feasibility, cost, and experimental constraints. Single-step models based on templates, sequence-to-sequence architectures, and graph neural networks have improved both forward prediction and retrosynthesis^1,4,35^. Hybrid planners that combine learned policies^36^ with symbolic search produce efficient multi-step routes and expose explicit trade-offs between search budget and route quality^3^. Evaluating planning algorithms is complex because route quality depends on budget, stopping criteria, route-cost models, and practical feasibility checks. Community efforts such as PaRoutes^12^ and Syntheseus^13^ demonstrate the value of shared route corpora and standardized metrics, though broader harmonization across single- and multi-step settings remains necessary.

**Fig. 1:**
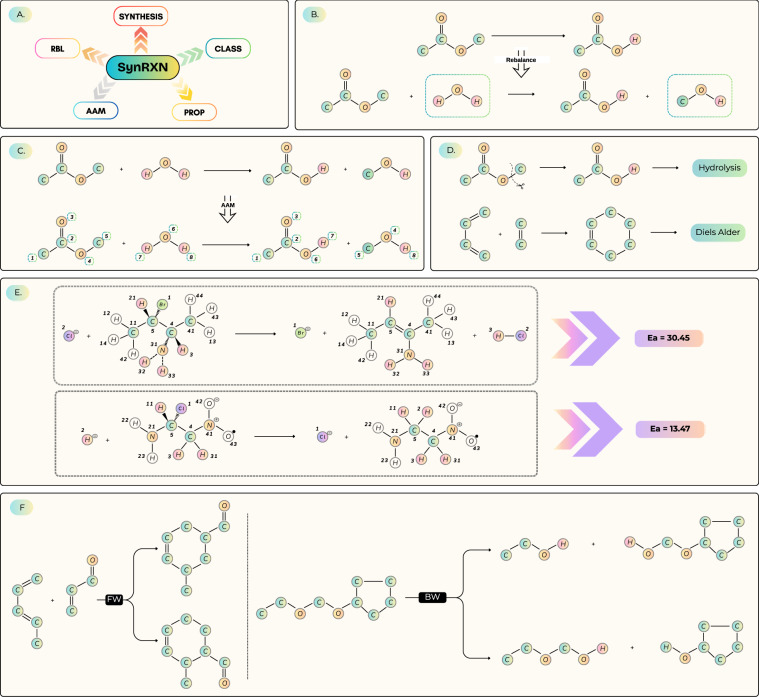
The SynRXN benchmark. (**A**) Benchmark suite overview. Individual tasks include (**B**) *reaction rebalancing*, (**C**) *atom-to-atom mapping*, (**D**) *reaction classification*, (**E**) *reaction property prediction*, and (**F**) *synthesis prediction*. All tasks provide curated datasets, predefined splits, and evaluation metrics.

These observations highlight a critical disparity: while the molecular machine learning community has flourished through standardized ecosystems like *MoleculeNet*^37^ and the *Therapeutics Data Commons*^38^, reaction informatics remains fragmented. Current studies often rely on bespoke subsets, inconsistent preprocessing, and opaque splitting strategies, rendering cross-paper comparisons nearly impossible. Although broad aggregation efforts like the *Open Reaction Database* (ORD)^5^ solve the data *access* problem, they do not provide the unified *benchmarking* layer necessary to rigorously evaluate the full CASP pipeline. To bridge this gap, we introduce SynRXN, a unified, FAIR (Findable, Accessible, Interoperable, and Reusable)^39^ benchmarking data resource (see Figure 1). SynRXN goes beyond simple data hosting by enforcing strict reproducibility: it supplies deterministic split functions that, when paired with our versioned manifest files and RNG seeds, recreate identical train-test partitions without the overhead of massive index files. SynRXN is complementary to raw corpora such as USPTO, and its goal is not to rehost reactions but to make model comparisons meaningful by standardizing task definitions, splits, and metrics across multiple upstream and downstream components, including rebalancing, atom-to-atom mapping (AAM), classification, property prediction, and single-step synthesis prediction. By providing dedicated benchmarks for both upstream and downstream tasks, SynRXN lets users evaluate each component under a consistent benchmark specification and report upstream quality alongside downstream predictive performance. SynRXN targets component-level, single-step benchmarking rather than multi step route planning, which depends on inventory and search protocol choices beyond the benchmark specification. We further provide held-out gold standards for sensitive upstream tasks like rebalancing and AAM. To avoid hidden assumptions, preprocessing choices are explicit and versioned, so users can adopt the reference specification for head to head comparison or substitute their own upstream methods while keeping the benchmark interface fixed. Available via PyPI (https://pypi.org/project/synrxn/) and Zenodo, SynRXN bundles standardized metrics, reference baselines, and CI-enabled regression checks. By codifying data hygiene and split transparency, SynRXN enables the first truly fair, head-to-head comparison of methods across the task landscape of reaction modeling.

## Methods

### Dataset construction

We assembled the SynRXN corpus from publicly available reaction repositories and community benchmarks, including USPTO patent-extracted reaction records^40^ and widely used derived sets such as USPTO_50K^41^, USPTO_MIT^42^, and USPTO_500^43^. A complete inventory of upstream sources and their assignment to SynRXN tasks is provided in the following sections and in the archived build manifest. We retrieved raw inputs from their original public release locations on GitHub and Zenodo and from supporting information associated with the source publications. For each component, we recorded the dataset identifier, resolved retrieval location, version or release tag when available, access date, file checksums, and redistribution license in a single manifest distributed alongside the curated release. Using this manifest, we executed deterministic build scripts to fetch inputs, validate checksums, and convert heterogeneous upstream formats into a unified reaction table schema. The curation pipeline applied molecular standardization, record-level validity checks, canonicalization to stable reaction identifiers, and deduplication, with stoichiometric rebalancing applied where required by the task definition. The entire pipeline can be reproduced from the version-pinned manifest by running the accompanying scripts in the script folder deposited with the Zenodo release and mirrored in the project repository. Redistribution follows the upstream licensing terms recorded per component in the manifest. Most components are provided under CC BY 4.0, while the SNAr subset retains CC BY 3.0 attribution requirements.

### Task datasets

#### Reaction rebalancing

Chemical reaction records mined from patent literature frequently lack stoichiometric fidelity, often omitting necessary inorganic reagents, solvents, or byproducts. Restoring mass balance in these records is critical for downstream modeling (Figure 1B), because missing reactants/products can yield under-specified (or atom-nonconserving) transformations and reduce the chemical executability of extracted retrosynthesis templates. In addition, mechanistic reaction modeling requires stoichiometrically faithful equations^44^. To construct a chemically robust *reaction rebalancing* benchmark, we derived targeted perturbation sets from the USPTO_50K corpus^35^, partitioning records into three specific modes of stoichiometric violation: MNC (Missing Non-Carbon), MOS (Missing One Side), and MBS (Missing Both Sides). Additionally, we curated a Complex set (1892 examples) from manually validated Golden and Jaworski collections^16,22^ to capture transformations involving significant skeletal rearrangements. The final compendium included: MNC (33147), MOS (12781), MBS (491), and Complex (1748) (Table 1a). Each subset was processed using SynRBL^15^, a hybrid rule- and graph-based algorithm for reaction completion. We retained only stoichiometric corrections resolved with a confidence of ≥90%.

**Table 1:** Rebalancing and atom-to-atom mapping benchmark datasets.

(a) Rebalancing task		
Dataset	Size	Reference
MNC	33147	^35^
MOS	12781	^35^
MBS	491	^35^
Complex	1748	^16,22^

(b) Atom-mapping task			
Dataset	Type	Size	Reference
Golden	Chem	1785	^19,22^
NatComm	Chem	491	^16^
USPTO_3K	Chem	3000	^19^
Recon3D	Bio	382	^45^
EColi	Bio	273	^46^

#### Atom-to-atom mapping

To benchmark *atom-to-atom mapping* (Figure 1C), we stratified the reaction corpus into two distinct domains: *synthetic chemical reactions* and *biochemical transformations*. The collection integrated diverse reference sets, comprising the Golden dataset (1785)^19,22^, the manually curated Jaworski subset (491)^16^, and a USPTO_3K partition (3000) sampled from USPTO_50K^19^, totaling 5,276 reactions. The biochemical partition aggregated metabolic data from Recon3D (382)^45^ and a validated EColi dataset (273)^46^, yielding 655 reactions. Prior to mapping, all records were subjected to a deterministic standardization and canonicalization procedure^47^. This pipeline enforced structural integrity by filtering anomalies (e.g., malformed SMILES strings or valence/charge violations) and ensuring uniform canonicalization. Topological fidelity is evaluated using two criteria: graph isomorphism of *Imaginary Transition State* (ITS) graphs^23,48,49^, and exact-match accuracy of canonicalized reaction SMILES verified via SynKit. Dataset statistics are detailed in Table 1b.

#### Reaction classification

*Reaction classification* requires mapping raw reaction inputs to predefined classes based on their structural or functional signatures (Figure 1D). We assembled a benchmark suite spanning multiple levels of granularity. The USPTO_TPL collection served as a fine-grained standard (1000 classes), annotated by deriving SMARTS templates from RXNMapper atom-maps^17,50^. For high-level categorization, we utilized the Schneider corpus (50 classes)^24^, which follows the hierarchical RSC reaction ontology. The USPTO_50K dataset^40^ provides two label sets: the legacy manual curation (10 classes)^35^ and a modern *structural relabeling* via SynTemp^23^. The latter enforces *center-specific isomorphism* and extends the reaction core to controlled radii (R0–R2) to capture subtle mechanistic variations. Finally, biochemical diversity is addressed via ECREACT^51^, which provides Enzyme Commission (EC) number hierarchies. To ensure rigorous evaluation, stratified splitting strategies were employed across all corpora to maintain *label density* across all folds (see Table 2).

**Table 2:** Reaction-classification benchmarks. Splits are stratified by reaction class and generated deterministically. The *Complete* column indicates whether reactions are fully specified or may be missing components.

Dataset	Size	Split ratio	Classes	Complete	Reference
Schneider_U	50000	9:1:40	50	No	^24^
Schneider_B	50000	9:1:40	50	Yes	^24,26^
USPTO_TPL_U	445115	8:1:1	1000	No	^50^
USPTO_TPL_B	445115	8:1:1	1000	Yes	^26,50^
USPTO_50K_U	50016	8:1:1	10	No	^35^
USPTO_50K_B	50016	8:1:1	10	Yes	^26,35^
SynTemp_R0	43441	8:1:1	143	Yes	^23,35^
SynTemp_R1	43441	8:1:1	356	Yes	^23,35^
SynTemp_R2	43441	8:1:1	680	Yes	^23,35^
ECREACT_1st	185734	8:1:1	7	No	^51^
ECREACT_2nd	185734	8:1:1	63	No	^51^
ECREACT_3rd	185734	8:1:1	175	No	^51^

#### Reaction property prediction

The *reaction property prediction* task (Figure 1E) targets the quantification of continuous chemical attributes. We assembled a comprehensive benchmark suite by aggregating data from public repositories (including Zenodo) and the literature. The suite encompasses *ab initio kinetics* datasets (e.g., B97XD3, LogRate), specific mechanistic classes (SNAr, SN2, E2), and high-throughput experimental results (e.g., RGD1). A significant portion of the data was sourced from the Heid collection and related works^31,52^, with additional datasets curated from the publications listed in Table 3. To ensure the integrity of the benchmark, all cleaning, standardization, and filtering operations were fully automated via SynRXN scripts. Datasets are provided in standardized formats containing either rxn (raw SMILES) or aam (atom-mapped SMILES) keys, mapped to specific property labels (e.g., “ea” for barrier height, “dh” for enthalpy).

**Table 3:** Reaction property datasets included in the SynRXN benchmark. The *H* column indicates whether the dataset includes explicit hydrogen atoms. The *Complete* column indicates whether reactions are fully specified (Yes) or may be missing components (No).

Dataset	Size	Split	AAM	H	Complete	Reference
B97XD3	16365	8:1:1	Yes	Yes	No	^58,59^
SNAr	503	8:1:1	No	No	Yes	^60^
E2SN2	3625	8:1:1	Yes	Yes	Yes	^31,32^
Rad6Re	31923	8:1:1	Yes	Yes	Yes	^31,61^
LogRate	778	8:1:1	Yes	Yes	Yes	^31,62^
Phosphatase	33354	8:1:1	Yes	No	Yes	^31,63^
E2	1264	8:1:1	Yes	Yes	Yes	^52^
SN2	2361	8:1:1	Yes	Yes	Yes	^52^
RDB7	23852	8:1:1	Yes	Yes	Yes	^52^
CycloAdd	5269	8:1:1	Yes	Yes^*^	Yes	^52^
RGD1	353984	8:1:1	Yes	Yes	Yes	^52^

^*^ Can be expanded to include explicit hydrogens (conversion available in our preprocessing scripts).

#### Synthesis prediction

The *synthesis prediction* task consolidates essential benchmarks for algorithmic single-step reaction prediction. We relied on three established subsets of the USPTO patent literature: USPTO_50K, the primary benchmark for template-based and template-free retrosynthesis^53^; USPTO_MIT, a high-volume corpus optimized for molecular transformer training^54^; and USPTO_500, a specialized dataset targeting reagent and catalyst inference^43^. These subsets are summarized in Table 4. Crucially, we recommend standardized, deterministic splits to resolve prevalent issues with benchmark comparability. Our accompanying evaluation suite standardizes reporting protocols, focusing on conventional top-*k* accuracy alongside structural similarity metrics.

**Table 4:** Reaction prediction corpora used in SynRXN.

Dataset	Size	Split	AAM	Task	Reference
USPTO_50K	50016	8:1:1	Yes	forward / backward	^19,35^
USPTO_MIT	479035	41:3:4	Yes	forward / backward	^54^
USPTO_500	143535	9:1:1.1	No	reagent prediction	^43^

## Data Records

**Fig. 2:**
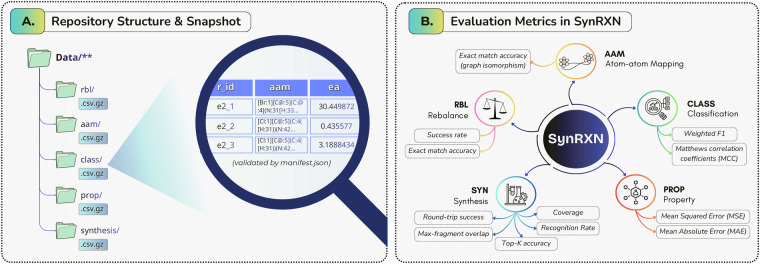
Overview of the SynRXN benchmark. (A) Data organization under the Data/ root, showing task-specific subdirectories and main tabular records. (B) Evaluation metrics employed for the different SynRXN tasks.

The SynRXN dataset is published as a machine-readable archive and source repository. Canonical releases are available from Zenodo^55^ and mirrored on GitHub at https://github.com/TieuLongPhan/SynRXN. Each release includes an authoritative manifest.json file that enumerates all data files under the project root, recording for each file its relative path, cryptographic checksum, row and column counts, column names, a short human-readable description, and file-level license information. This manifest serves as the primary source of provenance and is used by the build and verification scripts to check the internal consistency of each release. The top-level layout, file formats, and evaluation metrics are summarized in Figure 2. All data files in each release have an explicit license tag recorded in the top-level manifest.json under the license field.

All data records reside in the top-level Data/ directory, partitioned into task-specific subdirectories for the five benchmark tasks (Figure 2A): rebalancing (rbl), atom-to-atom mapping (aam), reaction classification (class), reaction property prediction (prop), and synthesis prediction (synthesis). Each dataset is provided as a gzip-compressed CSV file (.csv.gz) in UTF-8 with a single header row and one reaction per decompressed line. Missing values are encoded as empty fields. Datasets include metadata and predefined train/validation/test splits supplied either as an in-table split column or as companion split files, and may contain task-specific columns such as label, property, mapping-completeness or hydrogen-explicitness flags, and external-source identifiers. Exact file-level metadata (paths, checksums, row/column counts, column names and license tags) are recorded in the top-level manifest.json. Across all tasks, a small set of core columns is used consistently. The r_id column is a string that serves as a stable record identifier (e.g. uspto_00001), unique within each dataset. The rxn column contains the canonical, unmapped reaction SMILES, and aam holds the corresponding atom-mapped reaction SMILES when available. Downstream task labels are stored in either a label column (integer class codes for classification tasks) or a property column (floating-point reaction properties such as activation energies ea, enthalpies dh, or logarithmic rate constants lograte). Some datasets include additional, task-specific columns, for example flags indicating mapping completeness or hydrogen-explicitness, or identifiers linking back to external source corpora.

## Technical Validation

**Fig. 3:**
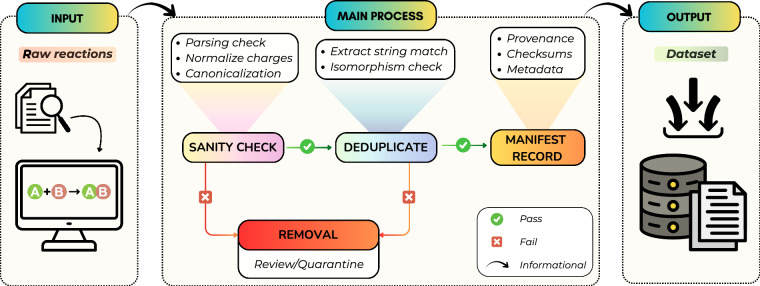
Technical validation workflow for the SynRXN benchmark.

Our goal is to provide dataset-level sanity checks and reproducible reference baselines that contextualize the released benchmarks, rather than to establish optimized or state-of-the-art task performance. To achieve this, raw reaction records were retrieved from their original sources and ingested without manual curation or augmentation. Each entry passed through an automated, deterministic canonicalization and chemical-sanity pipeline implemented in SynKit^47^, which is illustrated in Figure 3. The pipeline normalizes charges and valence states, standardizes aromaticity, and enforces a consistent SMILES canonicalization for all reactants, reagents, and products. We also perform automated duplicate detection during ingestion via (i) exact SMILES string matches and (ii) structure-level equivalence via isomorphism checks. Duplicate records identified by these checks are removed during ingestion; the dataset manifest records only the final counts so that the provenance and filtering outcome are reproducible. Records failing canonicalization or basic sanity checks (e.g. invalid SMILES, unparsable fields, impossible element/atom counts, or inconsistent valence) were excluded; no manual corrections were applied to excluded items. Finally, we provide reproducible baselines with fixed random seeds (default: 42) and fully specified preprocessing and training configurations. Unless stated otherwise, all models consume deterministic, canonicalized inputs, and all evaluations apply the same standardization and canonicalization to both predictions and curated references.

For the *reaction rebalancing* task, the *test* split is curated to provide ground truth: all candidate corrections proposed by SynRBL on the initial test pool were manually inspected, and only reactions that passed verification were retained. Because the final test set consists exclusively of these verified predictions, SynRBL serves as a reference baseline with 100% accuracy by design, reflecting a manually constrained subset of reliable labels rather than unconstrained automatic performance. For atom-mapped subsets, we retain atom maps from the original sources, and intentionally do not rebalance reactions because current atom-mapping models are typically trained or pre-trained on raw, often incomplete data. Mapped reactions are therefore only filtered by basic parsing and valence checks, such that invalid mapped SMILES or impossible valences are removed, while chemically plausible but stoichiometrically imbalanced reactions are kept, preserving the distributional characteristics of contemporary mapping corpora (baseline mapping accuracies for four tools across five held-out datasets are summarized in Table 5).

**Table 5:** Atom-mapping accuracy (%) across five datasets.

	EColi	Recon3D	USPTO_3K	Golden	NatComm
RXNMapper 0.4.1	72.53	48.69	93.53	87.43	87.58
Graphormer^*^	42.12	34.82	95.10	89.59	92.87
LocalMapper 0.1.5	69.96	50.79	97.77	89.08	92.67
RDTool 2.4.1	78.02	54.97	90.87	82.54	84.11

*Graphormer built with Cython 1.7.8.

For classification tasks, we report reference baselines using RXNFP and DRFP embeddings as fixed input features to a RandomForest classifier implemented in scikit-learn^56^. Performance is evaluated using repeated stratified cross-validation (5 repeats of 5-fold CV, i.e. 5 × 5 k-fold), following the statistical testing procedure of Ash *et al*.^57^. Stratified folds preserve class proportions and mitigate variance due to class imbalance. Primary evaluation metrics are the weighted *F*_1_ score^51^ and the multiclass Matthews correlation coefficient (MCC)^11^. Results for the stratified split are reported in Table 6.

**Table 6:** Reaction classification on stratified splits using DRFP and RXNFP embeddings with a RandomForest reference baseline. Values are mean  ± std; higher mean indicates better performance. Significance: NS (*p* > 0.05), * (*p* < 0.05), ** (*p* < 0.01), *** (*p* < 0.001), **** (*p* < 0.0001).

Dataset	Level	F1_weighted_*↑*			MCC*↑*		
		DRFP	RXNFP	*p*	DRFP	RXNFP	*p*
Schneider_U	—	**0.968** ± **0.002**	0.962 ± 0.002	****	**0.968** ± **0.002**	0.961 ± 0.002	****
Schneider_B	—	**0.953** ± **0.002**	0.936 ± 0.002	****	**0.952** ± **0.002**	0.935 ± 0.002	****
USPTO_TPL_U	—	**0.968** ± **0.002**	0.962 ± 0.002	****	**0.968** ± **0.002**	0.961 ± 0.002	****
USPTO_TPL_B	—	**0.953** ± **0.002**	0.936 ± 0.002	****	**0.952** ± **0.002**	0.935 ± 0.002	****
USPTO_50K_U	—	0.953 ± 0.002	**0.958** ± **0.002**	****	0.943 ± 0.003	**0.949** ± **0.002**	****
USPTO_50K_B	—	**0.966** ± **0.002**	0.952 ± 0.002	****	**0.958** ± **0.002**	0.941 ± 0.002	****
SynTemp	0	**0.952** ± **0.001**	0.920 ± 0.002	****	**0.954** ± **0.001**	0.927 ± 0.002	****
SynTemp	1	**0.940** ± **0.002**	0.897 ± 0.002	****	**0.943** ± **0.002**	0.903 ± 0.002	****
SynTemp	2	**0.913** ± **0.003**	0.737 ± 0.004	****	**0.907** ± **0.003**	0.714 ± 0.005	****
ECREACT	1	**0.977** ± **0.001**	0.905 ± 0.001	****	**0.966** ± **0.001**	0.862 ± 0.002	****
ECREACT	2	**0.964** ± **0.001**	0.857 ± 0.002	****	**0.961** ± **0.001**	0.846 ± 0.002	****
ECREACT	3	**0.949** ± **0.001**	0.840 ± 0.001	****	**0.947** ± **0.001**	0.835 ± 0.001	****

For datasets with numerical targets (e.g., yields, rates, or energies), we propagate the target values and their units exactly as provided by the original sources, each of which uses a single documented unit for the corresponding endpoint; any residual unit inconsistencies are thus inherited from the original data. To establish reference baselines for property prediction, we apply the same evaluation framework^57^ described for classification. The fixed embeddings are instead passed to a RandomForestRegressor^56^, with overall performance reported as mean  ± std. As a necessary exception for the exceptionally large RGD1 dataset, we use a single train-validation-test split evaluated over five random seeds. Empirical baseline performance for these regression tasks, reported as MAE and MSE^31^ in Table 7, indicates that the target distributions are learnable and do not exhibit obvious pathologies such as degenerate ranges or pervasive outliers.

**Table 7:** Reaction property prediction on random splits using DRFP and RXNFP embeddings with a RandomForest reference baseline. Values are mean  ± std; lower values indicate better performance. Significance: NS (*p* > 0.05), * (*p* < 0.05), ** (*p* < 0.01), *** (*p* < 0.001), **** (*p* < 0.0001).

Dataset	Prop	MAE *↓*			MSE *↓*		
		DRFP	RXNFP	*p*	DRFP	RXNFP	*p*
B97XD3	dh	19.838 ± 0.262	**19.323** ± **0.214**	****	649.814 ± 21.305	**599.521** ± **15.667**	****
B97XD3	ea	**14.617** ± **0.268**	15.324 ± 0.239	****	**376.803** ± **13.839**	396.723 ± 12.360	****
CycloAdd	act	**5.853** ± **0.157**	6.115 ± 0.157	****	**57.696** ± **4.071**	63.569 ± 4.261	****
CycloAdd	r	**11.790** ± **0.306**	12.081 ± 0.312	****	**227.691** ± **12.297**	236.306 ± 12.085	**
E2	ea	**3.247** ± **0.206**	7.377 ± 0.354	****	**20.067** ± **3.174**	91.161 ± 8.376	****
E2SN2	ea	**4.150** ± **0.126**	7.116 ± 0.133	****	**30.667** ± **2.074**	81.454 ± 2.857	****
LogRate	lograte	**1.054** ± **0.068**	1.077 ± 0.059	NS	**1.970** ± **0.284**	2.149 ± 0.355	**
Phosphatase	Conversion	**0.098** ± **0.001**	0.099 ± 0.001	****	0.019 ± 0.000	0.019 ± 0.000	****
Rad6Re	dh	1.126 ± 0.019	**0.908** ± **0.013**	****	2.585 ± 0.083	**1.612** ± **0.052**	****
RDB7	ea	30.136 ± 0.210	**18.812** ± **0.240**	****	1362.068 ± 16.817	**579.031** ± **15.282**	****
RGD1	ea	16.704 ± 0.074	**15.953** ± **0.032**	NS	495.386 ± 3.867	**453.876** ± **2.628**	NS
SN2	ea	**4.433** ± **0.161**	6.940 ± 0.234	****	**34.664** ± **2.426**	75.566 ± 4.393	****
SNAr	ea	**1.402** ± **0.158**	1.447 ± 0.139	NS	**4.348** ± **1.496**	4.355 ± 1.032	NS

## Usage Notes

The SynRXN loader supports three sources for programmatic access: Zenodo (stable, citable archive and recommended for publications), GitHub release tag (release artifacts) and GitHub commit (exact snapshot; may be unstable unless archived). Note that Zenodo queries can occasionally be delayed; enable GitHub fallback when immediate access is required.

We recommend using Zenodo for publication workflows (set source="zenodo" and provide version). Use source="github" for workflows driven by releases. For exact reproducibility, use source="commit" and provide the full commit_id; if those results are published, archive the snapshot (create a GitHub release or deposit on Zenodo) so it is citable. For tutorials, splitting strategies, and dataset construction reproducibility, see our documentation. (https://synrxn.readthedocs.io/en/latest/tutorials_and_examples.html).

## Data Availability

All data supporting this study are available in the SynRXN project repository (https://github.com/TieuLongPhan/SynRXN/) and as a versioned archive on Zenodo (SynRXN v0.0.8): 10.5281/zenodo.17672847

## Code availability

The SynRXN source code is available on GitHub: https://github.com/TieuLongPhan/SynRXN. Archived releases are available on Zenodo (SynRXN v0.0.8, DOI: 10.5281/zenodo.17672847). Comprehensive documentation is hosted at https://synrxn.readthedocs.io/en/latest/.
